# A Critical Review: Coupling and Synchronization Analysis Methods of EEG Signal with Mild Cognitive Impairment

**DOI:** 10.3389/fnagi.2015.00054

**Published:** 2015-04-20

**Authors:** Dong Wen, Yanhong Zhou, Xiaoli Li

**Affiliations:** ^1^School of Information Science and Engineering, Yanshan University, Qinhuangdao, China; ^2^The Key Laboratory for Computer Virtual Technology and System Integration of Hebei Province, Yanshan University, Qinhuangdao, China; ^3^Institute of Mathematics and Information Technology, Hebei Normal University of Science and Technology, Qinhuangdao, China; ^4^State Key Laboratory of Cognitive Neuroscience and Learning, IDG/McGovern Institute for Brain Research, Beijing Normal University, Beijing, China

**Keywords:** coupling, synchronization, EEG, mild cognitive impairment, Alzheimer’s disease

## Abstract

At present, the clinical diagnosis of mild cognitive impairment (MCI) patients becomes the important approach of evaluating early Alzheimer’s disease. The methods of EEG signal coupling and synchronization act as a key role in evaluating and diagnosing MCI patients. Recently, these coupling and synchronization methods were used to analyze the EEG signals of MCI patients according to different angles, and many important discoveries have been achieved. However, considering that every method is single-faceted in solving problems, these methods have various deficiencies when analyzing EEG signals of MCI patients. This paper reviewed in detail the coupling and synchronization analysis methods, analyzed their advantages and disadvantages, and proposed a few research questions needed to solve in the future. Also, the principles and best performances of these methods were described. It is expected that the performance analysis of these methods can provide the theoretical basis for the method selection of analyzing EEG signals of MCI patients and the future research directions.

## Introduction

Mild cognitive impairment (MCI), which acts as the transitory stage between normal aging and Alzheimer’s disease (AD), is often the main concern evaluating and diagnosing the early AD (Aarabi et al., 2008; Darvas et al., 2009; Knyazeva et al., 2010, 2013). As basic activity patterns between neurons or nerve clusters, coupling and synchronization become the important window through which people understand nervous system diseases including MCI and AD (Fell et al., 2001; Varela et al., 2001; Buzsáki and Draguhn, 2004). Relative to normal control (NC), the coupling and synchronization performance between different neurons or brain regions of MCI patients often show unusual behavior (Babiloni et al., 2006; Darvas et al., 2009; Vecchio and Babiloni, 2011; Knyazeva et al., 2013). EEG signals have the characteristic of coupling and synchronization, which make analyzing the abnormal state of MCI become possible from the angle of the EEG signals. The coupling pays close attention to the relationship between two EEG signals from different channels of single brain region or two brain regions, the synchronization more often appears in the relationship between two EEG signals from different brain regions or among multiple EEG signals from more brain regions (Fell et al., 2001; Womelsdorf et al., 2007).

Therefore, if we can calculate and analyze effectively the relationship of coupling and synchronization between different neurons or brain regions of MCI patients from EEG signal perspective, it will promote our understanding the predisposing mechanism of MCI and AD to a large extent. Many studies displayed the initial value of the coupling and synchronization analysis of EEG signals with application to evaluating MCI (Koenig et al., 2005; Babiloni et al., 2010; Dauwels et al., 2010b; Sweeney-Reed et al., 2012; Tóth et al., 2014). Therefore, the advantages and disadvantages of each coupling and synchronization method are the problems that at present we are in urgent need for understanding in-depth on diagnosis and evaluation of MCI. This paper reviewed in-depth, the various analysis methods of EEG signals of MCI patients from coupling and synchronization perspectives, and discussed the future research questions and trend.

## The Research Situation of EEG Signal Analysis Methods Used for Mild Cognitive Impairment Patients

### The coupling analysis methods

#### Methods Description and Evaluation

At present, the coupling analysis has become the focus of most concern in studying biological systems (Rosenblum and Pikovsky, 2001). The coupling between two EEG signals from different brain regions or two different electrodes is the object of much researchers concern, including of EEG signal coupling of normal subjects (Mizuhara and Yamaguchi, 2007; Cantero et al., 2009; Darvas et al., 2009) and subjects with diseases (Uhlhaas and Singer, 2006; Amor et al., 2009). In addition, many methods were used to analyze the coupling character between two EEG signals from different brain regions or two dissimilar electrodes of MCI patients, such as coherence (Moretti et al., 2008), mutual information (Liu et al., 2012), synchronization likelihood (SL) (Babiloni et al., 2006), Granger causality (Babiloni et al., 2009), and permutation conditional mutual information (PCMI) (Wen et al., 2014a).

For the method of coherence, it quantifies the linear correlation between two time series on frequency domain. Brassen et al. (2004) studied the MCI patients with depression by using the method, and the results showed that the MCI patients with depression were different significantly the NC in coherence strength between frontal and temporal area. However, the method does not consider the owner non-linear properties of signals.

For the method of mutual information, it calculates the own and joint probability density distribution of two time series, and quantifies the statistical independence between two time series by computing various entropies. Liu et al. (2012) analyzed the change related to the task in neural oscillation and connection between cerebral cortex of MCI patients and NC, and found that MCI patients were significantly different from NC in the neural oscillation strength and connection between parietal and occipital on theta frequency band. However, the computation of mutual information requires longer data, and shorter data are not enough to make the result of calculation have statistical significance.

For the method of SL, it is employed to calculate the degree of dynamic interactions between certain time series and another or multiple time series. Many studies displayed that the SL could be used for analyzing EEG signals of MCI patients: SL strength between EEG signals from frontal–parietal of MCI was lesser than NC on alpha1 frequency band, the SL strength between EEG signals from frontal–parietal of MCI was lesser than NC on delta frequency band (Babiloni et al., 2006), and the SL strengths of EEG signals of MCI were greater than NC on low alpha frequency band (8–10 Hz) (Pijnenburg et al., 2004). On the method of SL, the likelihood extent of time series patterns is calculated with statistical method, and this method determines which time series pattern is similar with other time series patterns according to threshold; however, the similarity is not considered in the decision-making process (Ahmadlou and Adeli, 2011), and it will affect the reliability of the method on diagnosing MCI to some extent.

Above most of the methods quantified the strength of coupling or coherence, were contributed to the in-depth study in analyzing EEG signals of MCI to a certain extent. Recently, many studies were trying to estimate the coupling direction between two EEG signals from different electrodes besides focusing on the coupling or coherence strength, such as Granger causality (Lungarella and Sporns, 2006), transfer entropy (Schreiber, 2000), conditional mutual information (Vejmelka and Paluš, 2008), instantaneous phases of interacting oscillators (Rosenblum and Pikovsky, 2001) and state space and phase-dynamics (Smirnov and Andrzejak, 2005), and so on. However, at present only Granger causality and its directed transfer function (DTF) were used to analyze the coupling direction or information flow of EEG signals from two electrodes of different brain regions of MCI (Babiloni et al., 2009; Dauwels et al., 2010b).

The Granger causality quantifies the degree of linear interdependence between different signals, and was often used in analyzing the linear model of EEG signals. The linear methods of Granger causality include partial directed coherence (PDC) and DTF, they belong to parametric method in accordance with multivariate auto-regressive model (Baccalá and Sameshima, 2001), and may describe the causality between multi-dimensional EEG signals on certain frequency band. The Granger causality methods achieved some valid results preliminarily in analyzing the EEG signals of MCI: the all frequency DTF of MCI decreased significantly in comparison with NC (Dauwels et al., 2010b), the direction index of information flow from parietal to frontal of MCI and AD decreased relative to NC, and especially the decrease became significant on alpha and beta frequency bands (Babiloni et al., 2009). The selection of order of multivariate auto-regressive model is difficult during estimating the parameters of multivariate auto-regressive model. Because lesser order affects the accuracy in estimating model parameters, bigger order can improve the accuracy and needed longer EEG signals to involve the calculation.

Recently, PCMI is a non-linear method, is used to estimate the coupling strength and direction of two time series from neural mass model, and also used to calculate the coupling strength and direction of time series of epilepsy and spike potential series (Li and Ouyang, 2010; Li et al., 2011). The studies showed that PMCI was superior to Granger causality in recognizing the coupling direction of unidirectional and bi-directional neural group. Meanwhile, Wen et al. (2014a) found the superiority of PCMI in analyzing the EEG signals of MCI in T2DM, and the results displayed that there exist differences between coupling strength or direction of two different brain regions of aMCI and control in T2DM on Alpha frequency bands. However, it remains to be further studied whether the method can be utilized for analyzing EEG signals of other types of MCI.

#### Problems to be Solved in the Future

According to above research status that presented the coupling analysis methods of two-channel EEG signals of MCI, we find that there exist a few key questions for further study in the future.

(1) It is urgently needed that know how to extract more meaningful features of EEG signals of MCI patients and dissect in-depth the interaction relationship between different EEG signals of MCI, in order to improve the computational accuracy of coupling strength.
(2) The vast majority of methods used to analyze EEG signals of MCI did not consider the calculation of coupling direction. Therefore, in the future we will need to explore new methods in order to calculate simultaneously the coupling strength and direction between different EEG signals of MCI.
(3) We need to improve the validity of statistical methods about coupling direction in the future, and change the present situation that existing methods relied on visual inspection or simple statistical method to estimate the main direction of information flow.

### The synchronization analysis methods

#### Methods Description and Evaluation

Many methods were used to estimate the synchronization strength of two time series and multiple time series, including of phase synchronization (Tóth et al., 2014), S estimator (Dauwels et al., 2010b), global synchronization (Koenig et al., 2005), stochastic events synchronization (Dauwels et al., 2010b), global synchronization index (GSI) (Cui et al., 2010; Lee et al., 2010), and global coupling index (GCI) (Wen et al., 2014b). And these methods were often applied to the studies analyzing the EEG signals of MCI and AD.

Phase synchronization refers to the interdependent relationship between instantaneous phases of two signals. Many studies showed that the phase synchronization strength of EEG signals of MCI was significantly different with NC (Sweeney-Reed et al., 2012; Tóth et al., 2014). The change appeared in synchronization features of MCI patients on delta and theta frequency bands before and after a year: the connect between frontal and temporal, frontal, and parietal decreased significantly, the function disconnection between different brain regions that are far apart from each other acted as a major feature of MCI patients (Tóth et al., 2014). There were the significant difference between MCI and NC on the time points and the degree of phase synchronization on theta and alpha frequency bands (Sweeney-Reed et al., 2012). In these studies, two methods extracting phase often were used, including wavelet transform and Hilbert transform (Quiroga et al., 2002; Pikovsky et al., 2003). Hilbert transform will appear obvious errors when computing the correlation between the phases of shorter EEG signals, and need longer EEG signals in order to estimate accurately the synchronization strength. Meanwhile, the phase synchronization method only extracts the phase feature of EEG signals and ignores other features.

Recently, S estimator was put forward, and it is a synchronization method based on state space, namely, it calculates the synchronization strength by analyzing the interdependent relationship among multiple signals in state space reconstruction domain (Carmeli et al., 2005). The S estimator was used to analyze the signals embedding dimension and time delay (Carmeli et al., 2005). Meanwhile, S estimator was preliminarily used for analyzing the EEG signals of MCI patients (Dauwels et al., 2010b), the research results of Dauwels et al. showed that the S estimator values of MCI patients were lesser significantly NC, and the Omega complexity of MCI patients were more than NC. Many researches showed that S estimator own good robustness and reliability in analyzing the data of model and real EEG signals (Quiroga et al., 2002; Knyazeva et al., 2013). However, S estimator does not consider adequately the effect of random and artifact component to analysis, and the accuracy of calculation has yet to be improved.

Global field synchronization (GFS) is another method measuring function synchronization, and employed to analyze the synchronization of multi-channel time series (Koenig et al., 2001). The method often focusses to the processing of EEG signals in frequency domain, and can estimate functional connection among multiple brain regions on different frequency bands (Koenig et al., 2005). Many studies showed that GFS could distinguish the EEG signals of MCI patients and NC on a few frequency bands (Koenig et al., 2005; Dauwels et al., 2010b). GFS belongs to a simple calculation between two normalized eigenvalues of the covariance matrix, and does not estimate accurately specific global synchronization strength. Meanwhile, GFS method acts as a simple multivariate method with quantifying synchronization of multiple signals, and first needs to calculate the synchronization correlation between two EEG signals. However, the GFS method is too simple to affect the accuracy of analysis for real EEG signals.

Dauwels et al. (2010a,b) proposed a method named stochastic event synchrony (SES) lately, and found that SES was better to analyze EEG signals of MCI patients relative to other methods. SES is characterized by calculating the interaction between certain events of EEG signals, extracted the point process from time-frequency representation of EEG signals, and quantified the similarity between point processes (Dauwels et al., 2009a,b; 2012). SES method showed the excellent performance when compared to the EEG signals of MCI patients and NC, and the SES values of EEG signals of MCI patients decreased significantly relative to NC (Dauwels et al., 2010a,b). However, the method was subject to limit of the number of channels, namely, the computation complexity increased greatly when the number of channels was added. The limitation affects the practicability analyzing actual multi-channel EEG signals.

Cui et al. (2010) proposed a new method named GSI, which improved the S estimator, in order to analyze the multi-dimensional neural series. Before calculate the GSI, the correlation coefficients between neural series were calculated with adopting mainly equal-time correlation method, which is a simple method measuring the linear correlation of two time series, does not estimate the non-linear correlation of two time series, and is also subject to the interference of noise to some extent. For all that, Lee et al. (2010) applied GSI to analyze EEG signals of AD, and found that GSI might act as biological identifier evaluating the cognitive decline of AD patients. However, the performance of GSI has not been reflected in analyzing the EEG signals of MCI systematically.

Recently, Wen et al. (2014b) improved the GSI method, and proposed a new method named GCI. The results showed that the synchronization strength based on GCI was less affected by the change of frequency bands relative to the other two methods, there existed more excellent performance on GCI method according to the change of coupling coefficient versus GSI and S estimator, and GCI was more sensitive than GSI and S estimator on distinguishing the synchronization strength of EEG signals from MCI and NC, especially in the Alpha frequency band. However, this method needed more time to calculate the global synchronization strength relative to GSI and S estimator.

#### Problems to be Solved in the Future

At present, there are the problems of two aspects, which are to be studied deeply in analyzing the synchronization of multi-channel EEG signals in the future.

(1) The profound relationship among two-channel EEG signals from multi-channel signals needs to be studied in-depth. Especially requires optimizing existing GCI method, decreases the time complexity of this method in the context of assuring accuracy, transforms the method into a reliable tool for estimating accurately the synchronization strength of multi-channel EEG signals.
(2) It is required to understand how compare the basic performance of several synchronization methods for analyzing multi-channel time series and real EEG signals. Generally, the criteria contains frequency band, coupling coefficient and signal to noise ratio for model data, and significant difference, correlationship, degree of accuracy, and time-consuming for clinical data.

### The principles and performances of coupling and synchronization methods

**Figure 1:**
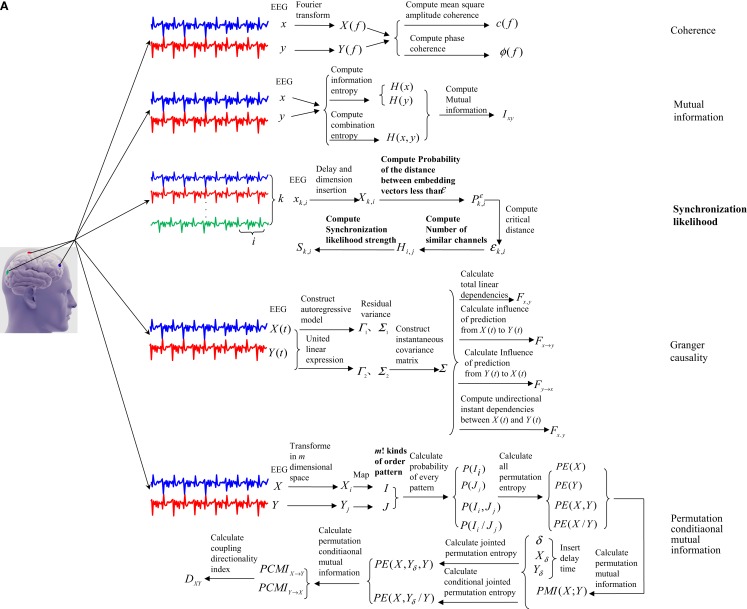

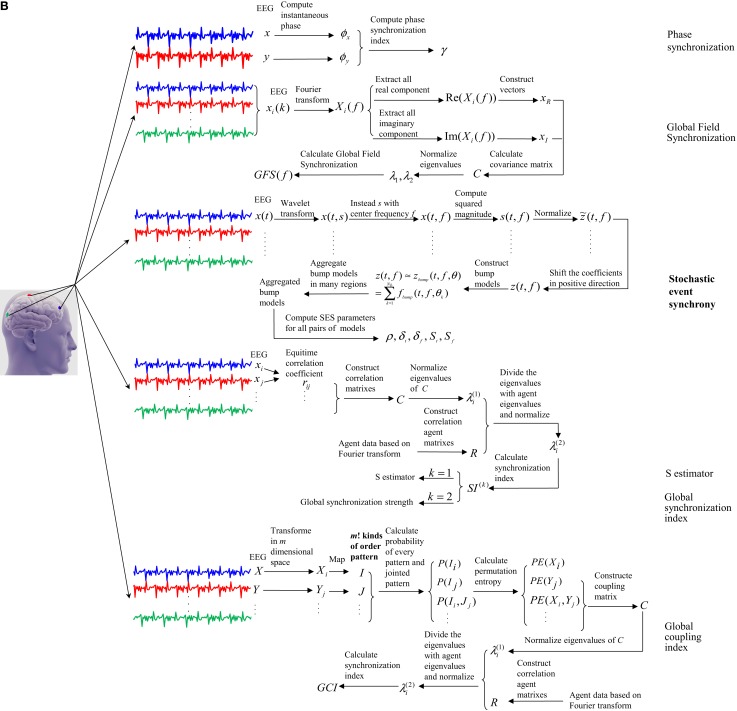
**The working pattern of five coupling and six synchronization methods with application to EEG signals of MCI patients**. **(A)** showed working pattern of five coupling methods, and **(B)** showed working pattern of six synchronization methods.

This paper summarized the principles and performances of the above coupling and synchronization methods. Figure 1 showed how the coupling and synchronization methods work in application to EEG signals of MCI patients. And Table 1 displayed the performance of the best indicators from these methods in analyzing EEG signals of MCI patients.

**Table 1:** **Performance of best indicators from coupling and synchronization methods in analyzing EEG signals of MCI patients**.

Methods	Accuracy	Sensitivity	Specificity	*P* value	Best indicators description
Coherence (Brassen et al., 2004)	–	88%	81%	0.004	Alpha band, from frontal to temporal
Mutual information (Liu et al., 2012)	–	–	–	*P* < 0.001	Theta band, all brain regions except in the occipital lobe
Synchronization likelihood (Babiloni et al., 2006)	–	–	–	*P* < 7 × 10^−6^ ~0.006	Delta band, F4–P4 of right fronto-parietal
Granger causality (Babiloni et al., 2009)	–	–	–	Alpha1: *P* < 0.00001	Alpha1 or Alpha2 bands, anterior–posterior
				Alpha2: *P* < 0.00001	
Permutation conditional mutual information (Wen et al., 2014a)	100%	100%	100%	*P* < 0.001	Alpha2 band, right temporal–parietal
Phase synchronization (Tóth et al., 2014)	–	–	–	0.002	Theta band, fronto-parietal in right or left hemisphere
S estimator (Wen et al., 2014b)	–	–	–	0.001	Alpha band, 10 channels including Fp1, Fp2, F7, C5, Fz, Cz, F8, C6, P3, P4
Global field synchronization (Koenig et al., 2005)	–	–	–	*P* < 0.0001	Alpha or Beta band, 19 channels of whole brain
Stochastic event synchrony (Dauwels et al., 2012)	87%	–	–	2 × 10^−5^	4–30 Hz, 21 channels of whole brain
Global synchronization index (Wen et al., 2014b)	–	–	–	0.008	Alpha band, 10 channels including Fp1, Fp2, F7, C5, Fz, Cz, F8, C6, P3, P4
Global coupling index (Wen et al., 2014b)	–	–	–	*P* < 0.00001	Alpha band, 10 channels including Fp1, Fp2, F7, C5, Fz, Cz, F8, C6, P3, P4

## Conclusion

In conclusion, the analysis of EEG signals of MCI patients from coupling and synchronization angles was proved to be the two important ways in evaluating and diagnosing MCI. Above studies considered the coupling and synchronization features of EEG signals from the activity of neurons and neural group, and analyzed the EEG signals of MCI patients from different sides. In view of the difference of these studies angles, which led the performance of the methods to show advantages and disadvantages, the results presented various difference, and this paper explained and analyzed the difference in detail. Meanwhile, the future researches need to be focused on the following aspects: exacts the deep features of EEG signals of MCI patients and analyze in-depth the interaction relationship between different EEG signals of MCI.

Explore many methods that calculate and count the coupling strength and direction between different EEG signals of MCI patients; studies in-depth the profound relationship between two-channel EEG signals, enhances the computational accuracy of global synchronization strength, and decrease time complexity; selects some more effective indicators to compare different coupling methods of two-channel EEG signals and synchronization methods of multi-channel EEG signals in all directions.

## Conflict of Interest Statement

The authors declare that the research was conducted in the absence of any commercial or financial relationships that could be construed as a potential conflict of interest.
